# C-Terminus Glycans with Critical Functional Role in the Maturation of Secretory Glycoproteins

**DOI:** 10.1371/journal.pone.0019979

**Published:** 2011-05-18

**Authors:** Daniela Cioaca, Simona Ghenea, Laurentiu N. Spiridon, Marioara Marin, Andrei-Jose Petrescu, Stefana M. Petrescu

**Affiliations:** Department Molecular Cell Biology, Institute of Biochemistry of Romanian Academy, Bucharest, Romania; Yale Medical School, United States of America

## Abstract

The N-glycans of membrane glycoproteins are mainly exposed to the extracellular space. Human tyrosinase is a transmembrane glycoprotein with six or seven bulky N-glycans exposed towards the lumen of subcellular organelles. The central active site region of human tyrosinase is modeled here within less than 2.5 Å accuracy starting from *Streptomyces castaneoglobisporus* tyrosinase. The model accounts for the last five C-terminus glycosylation sites of which four are occupied and indicates that these cluster in two pairs - one in close vicinity to the active site and the other on the opposite side. We have analyzed and compared the roles of all tyrosinase N-glycans during tyrosinase processing with a special focus on the proximal to the active site N-glycans, s6:N337 and s7:N371, versus s3:N161 and s4:N230 which decorate the opposite side of the domain. To this end, we have constructed mutants of human tyrosinase in which its seven N-glycosylation sites were deleted. Ablation of the s6:N337 and s7:N371 sites arrests the post-translational productive folding process resulting in terminally misfolded mutants subjected to degradation through the mannosidase driven ERAD pathway. In contrast, single mutants of the other five N-glycans located either opposite to the active site or into the N-terminus Cys1 extension of tyrosinase are temperature-sensitive mutants and recover enzymatic activity at the permissive temperature of 31°C. Sites s3 and s4 display selective calreticulin binding properties. The C-terminus sites s7 and s6 are critical for the endoplasmic reticulum retention and intracellular disposal. Results herein suggest that individual N-glycan location is critical for the stability, regional folding control and secretion of human tyrosinase and explains some tyrosinase gene missense mutations associated with oculocutaneous albinism type I.

## Introduction

N-glycosylation plays a pivotal role in glycoprotein maturation and functioning. Bulky N-glycans located at the surface of secretory and cell membrane glycoproteins molecules are mostly exposed to the extracellular space. These oligosaccharides serve multiple functions acting mainly as stabilizers and protective shields for the glycoprotein outside the cell and as recognition targets in adhesion and immune response modulation. Nevertheless, intracellular membrane glycoproteins facing the lumen of the subcellular organelles may be heavily glycosylated also, suggesting specific functions within the cell. The roles of these oligosaccharides in controlling intracellular processes such as folding, sorting and traffic of proteins have been extensively investigated (reviewed in [1], [2]). There is now clear evidence that N-glycosylation at distinct sites might perform different and multiple functions along the protein lifetime [3], [4], [5], [ and 6]. Evidence of possible selection pressure to preserve the N-glycosylation sites comes from the sequence comparison in many glycoproteins indicating the existence of conserved sets of N-glycosylation sites [7]. For example, it has been recently reported that several of the seven conserved N-glycosylation sites of the tyrosinase family proteins can perform distinct functions [8]. In the absence of a comprehensive analysis of human tyrosinase glycosylation, the study suggested that conserved sites may perform various functions.

The tyrosinase family of genes in vertebrates includes three related members encoding glyco-enzymes, tyrosinase, tyrosinase-related protein-1 (TRP-1) and tyrosinase-related protein-2 (TRP-2). These proteins determine melanin synthesis in pigment cells and are important players in mammalian pigmentation [9]. Human tyrosinase is a transmembrane glycoprotein with two copper ions in its active site and six or seven N-linked glycans exposed towards the lumen of specialized subcellular organelles called melanosomes [10], [11]. Tyrosinase regulates pigmentation by catalyzing the oxidation of L-tyrosine to L-DOPA which further initiates a series of oxidation and polymerisation reactions leading to the synthesis of melanin in melanosomes [12], [13]. Although tyrosinase polypeptide is synthesized at the endoplasmic reticulum (ER) and its maturation and folding occur within the ER lumen, the enzyme becomes fully functional only when it reaches the melanosomes. Hence, its intracellular transport starting from the ER and passing through the Golgi *en route* to melanosomes is tightly regulated at specific stages by multiple processes including the copper uptake and the N-glycosylation process [14], [15]. Tyrosinase polypeptide requires the acquisition of copper ions to become active and this step occurs in the Trans Golgi network and in melanosomes [15]. The absence of enzymatically active tyrosinase is the main cause of oculocutaneous albinism IA (OCA IA). Mutations leading to tyrosinase lacking N-glycans at N-337 or N-371 were reported in a number of OCA IA patients [16], [17]. Most OCA IA mutations lead to misfolded tyrosinase which is retained in the ER by the quality control (ERQC) and subjected to degradation by the ERAD pathway [18], [19].

A crucial role in the ERQC is played by calnexin (CNX) and calreticulin (CRT). These mannose binding lectins assist tyrosinase during its folding within the ER. We have previously shown that abolishing CNX association by inhibiting the N-glycosylation processing or impairing tyrosinase tethering to the membrane results in a misfolded protein unable to exit the ER [20], [21], [ and 22]. Entry of tyrosinase in the CNX and not CRT cycle is a decisive event in the formation of native disulfide bonds and polypeptide folding [23], [24]. CNX interacts with tyrosinase co-translationally following the attachment of its first two N-glycans during translation and translocation of the polypeptide within the ER [25]. However, whether the other five N-glycans are required for this interaction has not been systematically investigated.

Oligosaccharides could be specific recognition elements in the ERAD pathway. An important component of the ERAD pathway is EDEM 1, a mannosidase-like chaperone that regulates the extraction of misfolded polypeptide chains from calnexin cycle and sends them back to the cytosol for proteasomal degradation [26], [27]. Tyrosinase associates with EDEM1 during the ER maturation process, but data about the role of individual N-glycans in this process are rather scarce [28].

In this paper we investigate the role of conserved individual N-glycans in human tyrosinase maturation and intracellular traffic. To analyze the localization of N-glycans, a 3D model of the central active site unit of human tyrosinase was derived based on the X-ray crystallography data of *Streptomyces castaneoglobisporus* tyrosinase. We propose an N-glycan distribution covering two opposite sides of the molecule, occurring either in the close proximity or remote of the active site area. While two oligosaccharides located opposite the active site were specific CRT targets, glycosylation mutants analysis provided evidence that a pair of N-glycans covering the active side have been crucial for its ER export towards the secretory pathway. Moreover, while the N-terminus N-glycans are required for the early stages of the folding process, the C-terminus are conditional for completion of its post-translational productive folding.

## Materials and Methods

### Reagents, Cell Lines and Antibodies

Human embryonic kidney (HEK 293T) and A375 cells were obtained from the European Collection of Animal Cell Culture (Porton Down, UK). Cells were grown in DMEM, respectively RPMI 160 medium (EuroClone) containing 10% fetal calf serum (Biochrom), 50 units/mL of penicillin and 50 mg/mL of streptomycin (Invitrogen) and maintained at 37°C with 5% CO2. Rabbit anti-calnexin antiserum was a kind gift from Dr. J.J. Bergeron (McGill University, Canada) and the anti-ERGIC 53 antibody was from Dr. Hans-Peter Hauri (University of Basel, Switzerland). The mouse monoclonal anti-hemagglutinin antibody was from Santa Cruz Biotechnology (Santa Cruz, CA). T311 (NeoMarkers, Fremont, CA) is a monoclonal antibody IgG2a recognizing human tyrosinase. The cDNA encoding the mouse EDEM fused to an HA-tag in the pCMV-SPORT2 vector was a kind gift from Prof. K. Nagata and Dr. N. Hosokawa (Institute of Frontier Medical Science, Kyoto University, Kyoto, Japan). Kifunensine and lactacystin were from Toronto Chemicals, the other reagents were from Sigma.

### Tyrosinase N-glycosylation mutants

Mutant proteins were obtained based on the human tyrosinase cDNA expression plasmid, pTriEx-WT obtained previously [24]. Tyrosinase mutants lacking single or multiple N-glycosylation sites were obtained by changing the codon for Asn (AAT or AAC) from the glycosylation sequon to the codon for Gln (CAA), either using a site-directed mutagenesis kit (Clontech, USA) or by three-steps PCR method. Briefly, in this method, a set of reverse complementary oligonucleotide primers contained the base substitutions in the center of the primers. In the first and the second PCR reactions, these primers were paired with a 3′- and 5′-primer from the opposite end of the cDNA sequence, respectively. Then, the DNA sequences synthesized by the first two PCR reactions were used as template with the end primers for the third PCR reaction to generate the full-length recombinant DNA, which was cloned into *Bam*HI/*Xho*I site of pTriEx1.1.1 vector (Novagen, UK). The sliding glycosylation mutant construct was obtained based on tyrosinase mutant cDNA Δ7 with the following codon changed: Q378N (s7→378). For construction of the triple mutations Δ(1,2,3) (N86-N111-N161) and Δ(5,6,7) (N290-N337-N371), the mutagenic primers correspond to the middle glycosylation site; for the first two PCR reactions the mutants lacking single glycosylation site were used as template. Unglycosylated mutant Δ*all* was obtained with the mutagenic primers corresponding to the s4-N230 site based on the cDNA sequences of Δ(1,2,3) and Δ(5,6,7). Nucleotide sequence of all constructs was confirmed by sequencing.

### Transfection of Cells and Metabolic Labeling

Constructs were transiently transfected in HEK 293 and A375 cells. Semi confluent HEK 293 cells (50–70% confluence) 24 h post-seeding in 6-well dishes were used to transiently express tyrosinase cDNAs (3 µg of DNA/well) using polyethylenimine (PEI) solution (1 mg/mL, pH-8; 6 µL of PEI/well, Sigma) and 90% confluent A375 cells were transfected using Lipofectamine 2000 (Invitrogen) using 7.5 µL of Lipofectamine for 3 µg of DNA. Cells were analyzed 24 h after transfection. For metabolic labeling, transfected cells were starved in the cysteine – methionine free medium for 1 h, pulse labeled with 100–150 µCi of [^35^S] methionine/cysteine (MP Biomedicals) for 20 minutes and chased for the time specified. Immediately after chase cells were harvested in cold PBS with 20 mM N-ethylmaleimide (NEM). Cells were then lysed with CHAPS lysis buffer (50 mM HEPES buffer, pH 7.5, containing 2% CHAPS, 200 mM NaCl and 0.5% protease inhibitor mixture containing leupeptin, aprotinin, sodium EDTA, bestatin, 4-(2-aminoethyl) benzene sulfonylfluoride and E-46) for 1 h on ice. In some experiments lactacystin and kifunensine were added during starvation time and maintained during the pulse-chase period.

### Immunoprecipitation and SDS-PAGE

[^35^S]-labeled cell lysates were centrifuged, and supernatants were incubated with T311 antibodies (1∶50) overnight at 4°C, followed by the addition of 20 µl of protein A-Sepharose and further incubation for 1 h at 4°C. The slurry was washed three times with 0.5% CHAPS in HEPES buffer. Tyrosinase was eluted by boiling the slurry for 5 min in SDS sample buffer with 5% 2-mercaptoethanol or DTT. In some experiments, tyrosinase was eluted from protein with 1% SDS and the protein was resolved by SDS-PAGE under non-reducing conditions, i.e. in the absence of 2-mercaptoethanol. Calnexin co-immunoprecipitations were performed as described previously [24]. Briefly, lysates were immunoprecipitated with anti-calnexin, and the washed slurry was eluted with 1% SDS, diluted ten times with lysis buffer, and re-precipitated with T311 antibodies. The bound proteins were eluted in reducing conditions and resolved by SDS-PAGE. The gels were visualized by autoradiography. Relevant bands were quantified by scanning densitometry. In some experiments, 5 mM DTT was added to the cell culture medium at the end of the chase period. After 5 min cells were harvested in cold PBS containing 20 mM N-ethylmaleimide to alkylate the free SH groups and then lysed as described above.

### Immunofluorescence

HEK 293T cells were plated on cover slips and transfected with different constructs using the methods specified above. For some experiments cells were incubated with 20 mM nocodazole for 5 h before fixation. After 24 h the cells were rinsed with PBS and either fixed with paraformaldehyde (PFA) for 20 min at room temperature or fixed and permeabilized with methanol at −20°C for 5 min. After washing three times in PBS, cells were incubated with the primary antibodies T311 (1∶250), CNX (1∶200) and ERGIC-53(1∶250) diluted in PBS for 30 min at room temperature. Following three washes with PBS they were further incubated with the appropriate Alexa 594-conjugated and Alexa 488-conjugated secondary antibodies (1∶400) in PBS for 30 min at room temperature. Finally, cells washed three times with PBS were mounted on cover slips in Vectashield mounting medium (Invitrogen) and viewed with a Nikon Eclipse E 600 fluorescent microscope. Images were processed using Adobe Photoshop 5.0 software.

### Tyrosinase assay

The DOPA-oxidase assay measured the second major catalytic reaction of tyrosinase, the conversion of DOPA to DOPAchrome via DOPAquinone. This reaction was performed in 0.1M phosphate buffer, pH 6.8 and followed spectrophotometrically the chromogenic appearance of DOPAchrome from 1 mM DOPA at 475 nm [21]. Specific activity was measured using the Western blot quantitation of the actual amount of tyrosinase in the cell lysate and was calculated as the mean of three independent assays.

### Sequence analysis and Modeling

Tyrosinases were aligned with ClustalW [29]. An intrinsic disorder score for each position in the sequence was computed based on intrinsic disorder profiles generated with DisEMBL [30], IUPRED [31], RONN [32] and FoldIndex [33]. Consensus secondary structure propensity was determined based on SSPro [34], Jpred [35], GOR IV, SOPMA and HNN [36]. Other various physico-chemical sequence profiles were raised with in house software.

Human tyrosinase was modeled starting from *Streptomyces castaneoglobisporus*, pdb code 1wx2 [37]. Refined alignment and sequence to structure mapping was performed in Insight II and with Slide, a interactive threading software [38]. Modeling of sequence conserved regions and variable loop generation were performed with the Homology module in Insight II software from Accelrys. Protein model refinement consisted in iterations of local structure rebuilding followed by energy optimization and model quality assessment using MetaMQAP [39]. Energy optimization consisted in repeated rounds of energy minimization, and local and global simulated annealing performed with Discover in Insight II using the cvff force field.

The glycan moiety was modeled with Glyco-Pack, a software for glycoprotein structural analysis and modeling [40] and with Amber [41]. Five GlcNAc2Man7 N-glycans were attached *in silico* to the model of human tyrosinase protein core at glycosylation sites s3:N161, s4:N230, s5:N290, s6:N337 and s7:N371 in configurations consistent with the most populated conformer derived from the SAGS data base of experimental glycoprotein structures [42]. The glycan attachment, conformational search, and clash analysis were performed with Glyco-Pack and the glycoprotein structure was further optimized in Amber 10, using Glycam 06 and ff99sb force fields [43].

## Results

### Human tyrosinase 3D modeling

**Figure 1:**
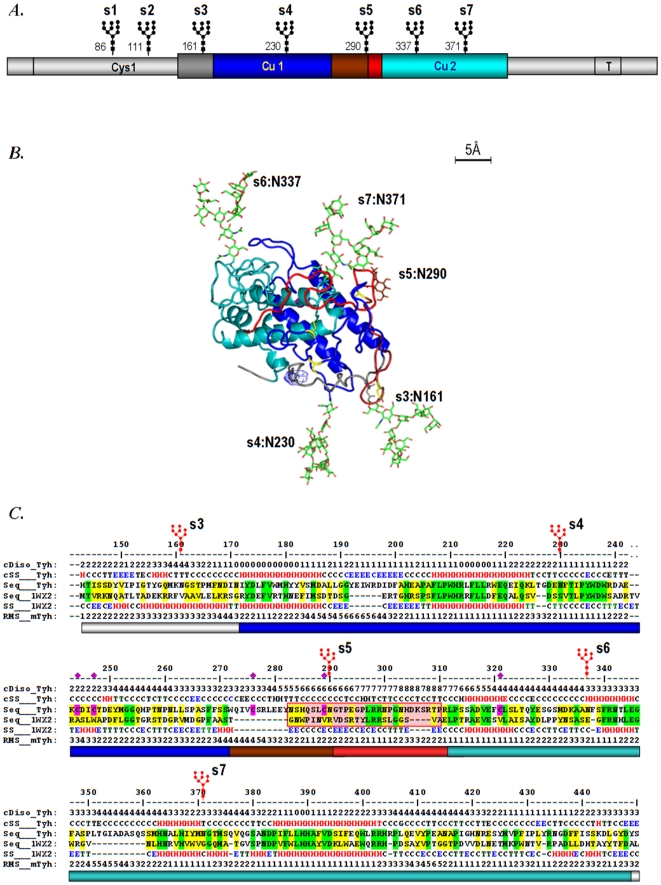
Restricted 3D model of human tyrosinase. **A.** Location of the active site region of human tyrosinase (hT) modeled starting from *Streptomyces castaneoglobisporus* tyrosinase (sT): extended copper domains Cu A and Cu B are shown in blue and cyan, and the linker is in dark and light red according to its predicted flexibility. **B.** The 3D model of hT with its maximal error scale (5 Å). Cysteines are in yellow and the position in space of the unoccupied s5 is shown by one red sugar unit. **C.** Sequence alignment of hT with sT: *cDiso* - shows the consensus of predicted hT disorder, *cSS* - shows the consensus of predicted hT secondary structure and the *RMS* - shows the predicted departure of the model from an optimal path, in Angstroms.

Vertebrate tyrosinases are type I transmembrane proteins of ∼530 aa length. Their sequences were historically divided in two copper domains, Cu A and Cu B, preceded and interposed respectively by two so called cysteine rich domains, Cys1 and Cys2, followed by a C-terminal transmembrane and cytosolic domain, (Fig. 1) [44].

Sequence alignment reveals that the central region of human tyrosinase (hT: aa 142–450) overlaps with the prokaryal tyrosinase from *Streptomyces castaneoglobisporus* (sT) (Fig. 1). This roughly excludes downstream the Cys1 domain, and upstream the TM and the cytosolic domains. Within the overlapping region the overall identity between the target and the template is over 25%, and the homology is over 55%. Moreover the similarity is unevenly distributed and it clusters into two main sub-regions of 113aa and 154aa respectively, with over 30% identity and 60% homology, which are connected by a stretch of 36 aminoacids in hT (aa272–308), with 14 aa longer that its counterpart in sT. This makes the region aa142–450 of human tyrosinase to fall into the generally accepted limits of homology modeling [45].

Model refinement of inserts: aa192–196, aa273–282, aa305–308 and of the N-terminus region within aa142–170 which consisted into bringing locally the structure in compliance with the hT secondary structure prediction, results in an increase of the overall model accuracy from 3.17 Å to 2.48 Å root mean square deviation (rmsd), with the global distance test total score raising from GDT-TS = 54.14 to 62.91. The largest local deviation of the model from an optimal alpha carbon path does not exceed 6 Å rmsd and reach its maximum in the insert aa192–196, a region that does not inflict on the placement of N-glycans, nor onto the location of cysteine residues in the Cys2 domain (Fig. 1).

All major intrinsically disorder predictors - FoldIndex, disEMBL, IuPred, RONN etc indicate a high propensity for flexibility of the hT sequence in the region aa290–315, while the DLP predictor [46] indicate a high linker propensity in the region aa283–317. In this regard the five cysteine residues do not fall into a well defined structural unit. C244, C247 belong to the extended Cu1, C321 belongs to the extended Cu2, while C276 and C289 are located within the linker. Moreover, the model suggests that the five cysteine residues are unlikely to form disulfide bonds between them, and hamper by this the dynamic disorder propensity of this region. For example the distance between the closest cysteine residues C276 and C247 is over 19 Å while the cumulated model rmsd is less than 9 Å. It is therefore more likely that either some of these cysteine residues are involved in disulfide bridges with cysteine residues from the Cys1 domain, or they might have only a regulatory protonation role in the melanosomal environment.

The model hosts also the last five out of seven glycosylation sites of human tyrosinase, s3:N161, s4:N230, s5:N290, s6:N337 and s7:N371. In sequence these are evenly distributed (Fig. 1A). However, in space they cluster into two pairs: the C-terminal sites s6 and s7 within the active site region, and s3 and s4 on the opposite side of the protein. The fifth site, s5:N290, is located into the linker region, right at the beginning of the most disordered stretch of human tyrosinase, aa290–315, yet this sequon is followed immediately by a proline in position 293 (N+3) which reduces the probability of occupation. The close distance of C-terminal N-sites s6 and s7 to the active site suggests that their occupation might influence the local structure and hence the enzymatic activity more than it does occupation of N-sites s3 or s4.

Assessment of the Cys1 sequence properties failed to result into a reliable 3D model. Secondary structure predictors pinpoint along Cys1 four beta strands followed by one helix with 3 or 4 turns toward its C-terminal end. Due to this mainly beta architecture and to the large number of cysteine residues and their pattern, the fold recognition predictors such as Phyre [47] classify the Cys1 domain as belonging with high probability either to the EGF/laminin (2.10) or to the IG-like (2.60) architectures, according to CATH [48]. The homology with the closest templates, 1toz, 2gy5, 2h9e is however less than 15%, at the limit of statistical noise, and in addition the target-template alignment do not cover the helical region as well, hence a plausible model of Cys1 could not be raised starting from these templates. Nevertheless, we can predict that Cys1 may act as a lid located on top of the active site unit, in a configuration more or less similar to that of the caddie protein which co-crystallizes with the sT template.

The remarkable conservation of the active site area explains the identity of function between eukarial and prokarial tyrosinases with eukarial enzyme acquiring during evolution specific molecular determinants such as the Cys1 domain, the TM domain and the seven N-linked glycans. To further investigate the role of N-glycans in human tyrosinase function we constructed and characterized tyrosinase glycosylation mutants.

### Enzymatic activity of the N-glycosylation mutants

Single glycosylation mutants (Δ) at all seven sites, N86, N111, N161, N230, N290, N337 and N371, two triple mutants Δ(1,2,3) and Δ(5,6,7) and the unglycosylated mutant Δ*all* were constructed and analyzed in terms of site occupancy, N-glycan processing and enzymatic activity. The N-glycosylation mutants were expressed in a previously validated mammalian system using kidney embryonic cells [23], [24], [ and 49]. In this system, transiently transfected wild type tyrosinase is translocated into the ER where the glycosylated polypeptide chain folds in the presence of calnexin/calreticulin into an export competent protein that exits the ER.

**Figure 2:**
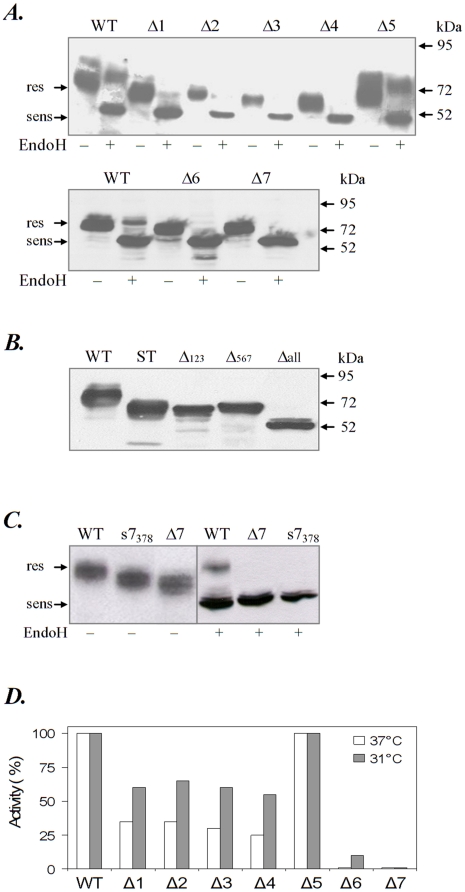
N-glycan position determines the maturation of tyrosinase mutants. HEK 293 cells were transiently transfected with **A**) wild type tyrosinase (WT), Δ1–Δ7 single glycosylation mutants, **B** Δ(1,2,3), Δ(5,6,7), Δ*all* mutants and **C**)**WT**, Δ7 and s7_378_ mutants. 24 h post-transfection cell lysates were EndoH digested and immunoblotted with anti-tyrosinase antibody. ST-soluble tyrosinase (69 kDa) was used as molecular mass control; res, the EndoH-resistant secreted forms; sens, the high mannose ER-retained forms. (**D**)Enzyme activity of the mutants was assayed by measuring their DOPA oxidase activity at 37°C and 31°C. Data are expressed as percentages of the wild type tyrosinase specific activity and are the averages of three independent experiments.

Six out of seven sites were occupied, as seen from the decrease in mobility caused by the ablation of a consensus site in the single mutants shown in Fig. 2A and B. No change in mobility was observed for the Δ5 mutant expressed either in HEK cells (Fig. 2A) or in the A375 melanoma cells (supplemental Figure S1) suggesting that this sequon is rarely occupied in human tyrosinase. The two triple mutants displayed slightly different molecular masses corresponding to three (Δ(5,6,7)) and four (Δ(1,2,3)) glycans, respectively (Fig. 2B). The unglycosylated mutant migrated at 60 kDa, corresponding to the polypeptide mass (Fig. 2B). The faint band migrating above the polypeptide could represent a minor molecular species with uncleaved signal sequence.

We further determined the N-glycan processing of the mutants from EndoH sensitive (*sens*) structures of high mannose/hybrid type characteristic to the ER/early Golgi to complex type structures EndoH resistant (*res*) acquired within the Golgi. Expression of wild type tyrosinase in HEK cells yielded an EndoH sensitive and an EndoH resistant population (Fig. 2A). This is not surprising, since it has been previously reported that tyrosinase folding and maturation is rather inefficient resulting in less than 50% of the newly synthesized polypeptide being processed to EndoH resistant forms [24]. Only the Δ5 mutant behaved similarly, while a very faint EndoH resistant form was observed for Δ1 (Fig. 2A). All the other mutants were EndoH sensitive (Fig. 2 A). This indicates that all six N-glycans are required for the normal processing of human tyrosinase. Some minor stringency of s1 N-glycan may account for the small population of Δ1 molecules that are able to mature properly and leave the ER.

To explore the role of the conserved position of the N371 we further changed the location of s7 in sequence by constructing a double mutant, N371Q/Q378N. The new selected location was largely accessible, with no glycan-protein collisions or albino mutations. As shown in Fig. 2C, the mobility shift of the double mutant as compared to Δ7 indicates that the created sequon N378 has been glycosylated within the cell (Fig. 2 C, left panel). However, the wild type enzymatic activity could not be rescued (data not shown) and the mutant remained Endo H sensitive (Fig. 2C, right panel). There is a remarkable stringency for glycosylation at N371 which is actually the most conserved site in all species and all members of tyrosinase protein family. Work is in progress to discriminate between various scenarios explaining this result and will be described elsewhere (manuscript in preparation).

We have previously shown that wild type tyrosinase trafficking through the secretory pathway yields a functional protein with DOPA oxidase activity [20]. The assay of tyrosinase function revealed that the DOPA oxidase activity of the mutants was completely abolished in the mutants Δ6 and Δ7, dramatically reduced in the mutants Δ1, Δ2, Δ3, Δ4 and unchanged in the Δ5, which suggests different functions for the individual N-glycans of tyrosinase (Fig. 2D). The loss of enzymatic activity of the Δ6 and Δ7 mutant correlated with the disruption of their maturation process. Likewise the partial processing to Golgi structures of wild type tyrosinase and Δ5 mutant correlates with their similar enzymatic activities. The residual activity of the EndoH sensitive immature Δ2, Δ3 and Δ4 mutants presented could be due to post-lysis activation occurring in the presence of DOPA [13], which may suggest that these immature polypeptides are not terminally misfolded. To investigate whether the lack of enzymatic activity was due to folding defects of the polypeptide we further tested the temperature sensitivity of the mutants. Since some misfolded tyrosinase mutants may fold and recover their enzymatic activity below 37°C [24] we have expressed the mutants in cells cultivated at 31°C. Mutants Δ1, Δ2, Δ3 and Δ4 showed a marked increase of their enzymatic activity after 24 h cultivation at lower temperature. These data confirm that deletion of any of the four N-terminal N-glycans induces temperature-sensitive folding defects in tyrosinase. In contrast, Δ6 displayed a rather low recovery and Δ7 was completely inactive, indicating that the two mutants cannot be rescued by slowing down the folding process (Fig 2D).

In brief, ablation mutant data show that human tyrosinase has six N-linked glycans with the s5 sequon unoccupied at least in the two cell lines tested. The two mutants lacking the N-glycan at the s6 and s7 C-terminal sites show impaired processing and no activity. The remaining mutants show intermediate activity, ranging from 25% to 30%. In addition, these four mutants are temperature sensitive, in contrast to the couple of C-terminal mutants that are either low responsive or irreversibly inactive even at the permissive temperature.

### ER retention of the N-glycosylation mutants

**Figure 3:**
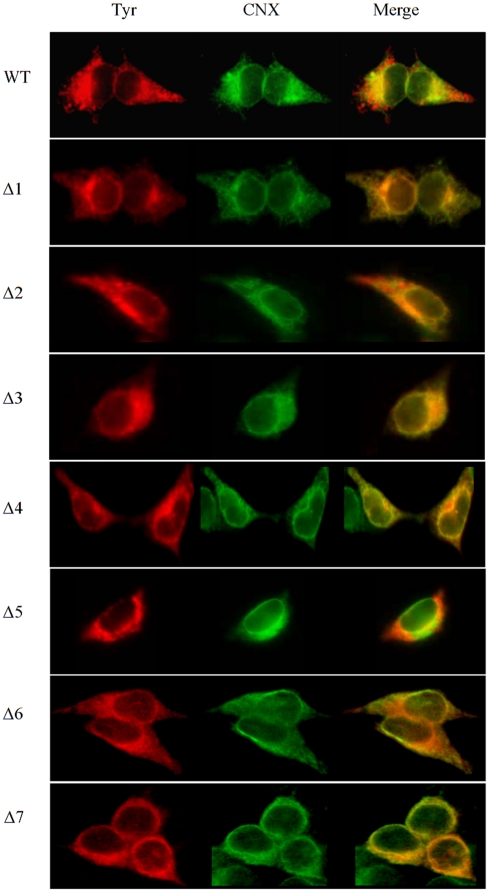
ER localization of N-glycosylation mutants. Cells were transiently transfected with WT and the single mutants Δ1–Δ7. 24 h post-transfection cells were fixed and permeabilised with methanol and processed for anti- CNX (CNX) and anti-tyrosinase (Tyr) immunofluorescence. The yellow/orange color in merged images indicates where tyrosinases co-localized with CNX in the ER. A massive overlapping with CNX is present for all tyrosinases, with some non-overlapping red and green fluorescence for the WT and Δ5, attributable to non-ER localization.

The EndoH digestion experiments showed that excepting wild type and Δ5 that are processed to significant populations secreted to the Golgi, the other mutants are mainly EndoH sensitive, which indicates high mannose ER structures, but also hybrid type N-glycans occurring within the early Golgi. To confirm the ER retention or Golgi transport of the glycosylation mutants we performed co-localization of the mutant tyrosinases with the ER marker calnexin in immunofluorescence experiments. Wild type tyrosinase and the Δ5 mutant co-localized only partially with calnexin and displayed a cytoplasmic pattern consistent with an intracellular vesicular distribution (Fig. 3). In contrast, all the other mutants largely co-localized with the ER marker calnexin, confirming their ER localization (Fig. 3). These data complete the N-glycan processing analysis indicating that wild type and the Δ5 mutant were partially processed to mature proteins and secreted from the ER. while all the other single mutants were mainly retained within the ER at 37°C. At least for the archetypal mutants, i.e. Δ5 and the pair Δ6 and Δ7, the ER export process was found to be a pre-requisite for the enzymatic activity, indicating possible differential functions for individual N-glycans in tyrosinase traffic.

### Association of tyrosinase mutants with calnexin

**Figure 4:**
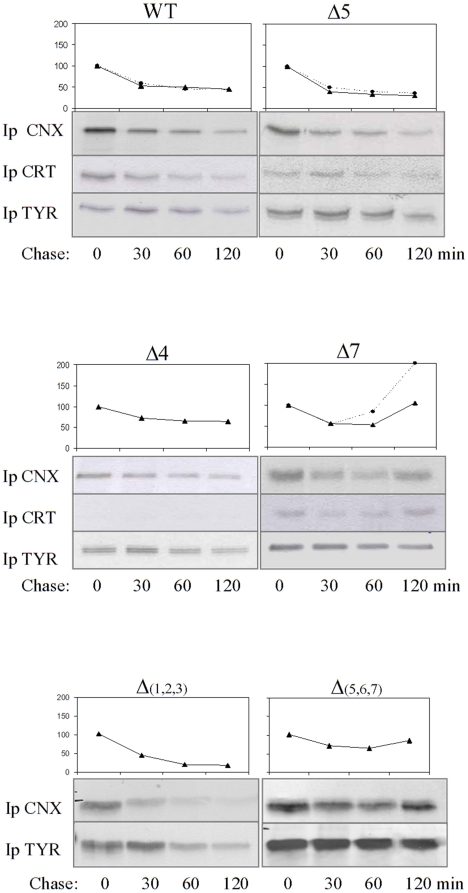
Association of the N-glycosylation mutants with calnexin and calreticulin. Cells were transiently transfected with WT, Δ4, Δ5, Δ7, Δ(1,2,3) and Δ(5,6,7) mutants. 24 h post-transfection cells were pulsed for 20 minutes and chased for 0, 0.5, 1, 2 hours. Cell lysates were sequentially precipitated with anti-calnexin or anti-calreticulin and T311 antibodies (Ip CNX, Ip CRT). To determine the total amount of tyrosinase an aliquot of the lysate was precipitated with T311 antibodies (Ip TYR). Samples were subjected to 10% SDS PAGE and autoradiographed. One of at least two representative experiments is shown. The ratio CNX (▴) and CRT (•) bound tyrosinase/total tyrosinase over time has been plotted.

We and others have previously shown that human tyrosinase requires CNX/CRT association for proper folding within the ER [24], [25]. The co- and post- translational interaction is transient with a progressive decrease during tyrosinase ER maturation accompanied by a parallel increase in the Golgi secreted protein. To analyze the role of individual glycans in this lectin type association with calnexin during tyrosinase folding, we made use of the single N-glycosylation mutants and of the two triple mutants. The metabolically labeled cells were first immunoprecipitated with antibodies against CNX or CRT and tyrosinase was recovered from the complexes by a second immunoprecipitation with anti-tyrosinase antibody (Fig. 4 and supplemental Figure S2).

Wild type and Δ5 mutant associated with CNX/CRT in a time-dependent manner with a maximal association at the beginning of synthesis. These time course interactions indicate that the polypeptide associates with the lectin chaperones during the folding process and gradually dissociates from the native molecules remaining associated only with the incompletely folded ones. In contrast, Δ7 associated with CNX/CRT with a different kinetics (Fig. 4) since the amount of mutant bound to the lectin increased during the maturation process, implying that this mutant is unable to fold properly and it is retained within the cycle before degradation. The mutants Δ1, Δ2, Δ3, Δ6 (supplemental Figure S2) and Δ4 (Fig. 4) showed less prolonged interactions with CNX, indicating that they were retained in the CNX cycle less than the Δ7 mutant. However, the patterns of association with CNX/CRT are variable, with the Δ7 terminally misfolded mutant displaying the longest retention time. For example, Δ2 was closest to WT in terms of CNX interaction. Since this mutant interacted longer with CRT, the ER retention was driven here by CRT rather than CNX. Furthermore, retention in CNX cycle is not a simple add up function with the number of ablated glycan sites, as shown by the triple mutants Δ(1,2,3) and Δ(5,6,7). For example, the mutant Δ(1,2,3) dissociated with a rate comparable with WT while mutant Δ(5,6,7) had a prolonged interaction with CNX, similar to Δ7 (Fig. 4). The fast dissociation rate of the Δ(1,2,3) could be due to its delivery to the kifunensine dependent degradation pathway which was markedly different for the two ER retained triple mutants, as shown below.

Interestingly, little association if any with CRT was found for the Δ4 (Fig. 4) and Δ3 mutants (supplemental Figure S2) up to 2 h of chase. This indicates that not all N-glycans of newly synthesized tyrosinase transiently associate with CRT and that s3 and s4glycans are situated in a region of the molecule accessible to the lectin CRT.

### Disulfide bonds formation during the folding of the Δ7 mutant

**Figure 5:**
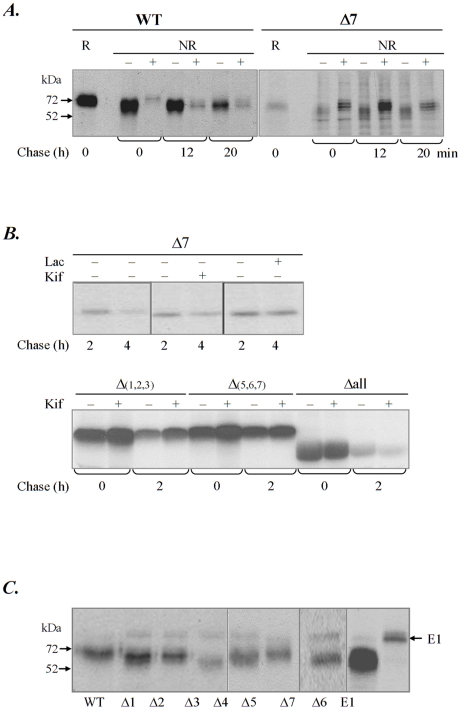
Misfolded mutants are degraded by ER associated degradation pathways. **A.** Cells were transiently transfected with WT and the Δ7mutant. 24 h post-transfection cells were pulsed for 8 min. and chased for 0, 12 and 20 min. In some samples DTT was added with 5 min. before harvesting. Cell lysates were immunoprecipitated with T311 antibodies and resolved by non-reducing (NR) and reducing (R) 7.5% SDS PAGE. The result was visualized by autoradiography. **B.** Cells were transiently transfected with WT, Δ7, Δ(1,2,3), Δ(5,6,7) and Δall. 24 h post-transfection they were pulsed for 20 min. and chased for the time indicated in the absence/presence of proteasomal inhibitor lactacystin (Lac) or mannosidase inhibitor kifunensine (Kif). **C.** Cells were transiently co-transfected with WT, Δ1–Δ7 mutants and EDEM1 (E1). 24 h post-transfection cells were pulse-chased for 20 min. Cell lysates were precipitated with T311 antibodies. All mutants tested co-immunoprecipitate the EDEM1 protein. One of at least two representative experiments is shown.

We next examined the disulfide bond formation in wild type tyrosinase versus the Δ7 mutant by assessing the accessibility of these bonds to DTT. Transfected cells were pulsed for 8 min., chased for 0, 12 and 20 minutes and incubated or not with 5 mM DTT before harvesting. We have previously found that this DTT concentration cleaves only exposed disulfide bonds whereas completely folded proteins with buried disulfides are unaffected [23]. Peleted cells were alkylated with 20 mM N-ethylmaleimide to prevent further oxidation, lysed and immunoprecipitated with T311 antibody. As previously reported, wild-type tyrosinase became DTT sensitive at about 12 min chase, as seen from the slight mobility shift of the DTT treated samples at this chase-point. A similar increase in the mobility shift was found at 20 min chase (Fig. 5A). In contrast, the mutant protein Δ7 followed an unproductive folding pathway characterized by the formation of intermediates displaying disulfide bonds accessible to DTT for all the chase times. This experiment proved that s7 glycan is critical for the folding process of human tyrosinase since the Δ7 mutant was unable to adopt a three-dimensional conformation stabilized by native disulfide bonds.

### ER associated degradation of recombinant tyrosinases

We have further investigated the effect of the proteasomal inhibitor lactacystin and of the mannosidases inhibitor kifunensin on the degradation of tyrosinases by pulse chase and immunoprecipitation. As shown in fig. 5B, treatment with both inhibitors determined the accumulation of the Δ7 single mutant in the ER. Similar data were found for wild type tyrosinase (Fig. 5B) confirming previously reported data and for the other single mutants (data not shown), thus confirming that all tyrosinase N-glycosylation mutants are ERAD substrates with proteasomal degradation. Interestingly, while the triple mutant Δ(5,6,7) has a reduced inhibition of degradation in the presence of kifunensine, the triple mutant Δ(1,2,3) shows a more pronounced accumulation of the polypeptide. As a control, we used the non glycosylated mutant whose degradation at 2 h of chase could not be rescued by kifunensine treatment (Fig. 5B).

To analyze the interaction of EDEM1 with the mutants, we have overexpressed EDEM1 and the mutants and immunoprecipitated tyrosinase from the cell lysates with anti-tyrosinase antibodies. As shown in Fig. 5C all single glycosylation mutants and wild type tyrosinase interacted with EDEM1 (Fig. 5C).

**Figure 6:**
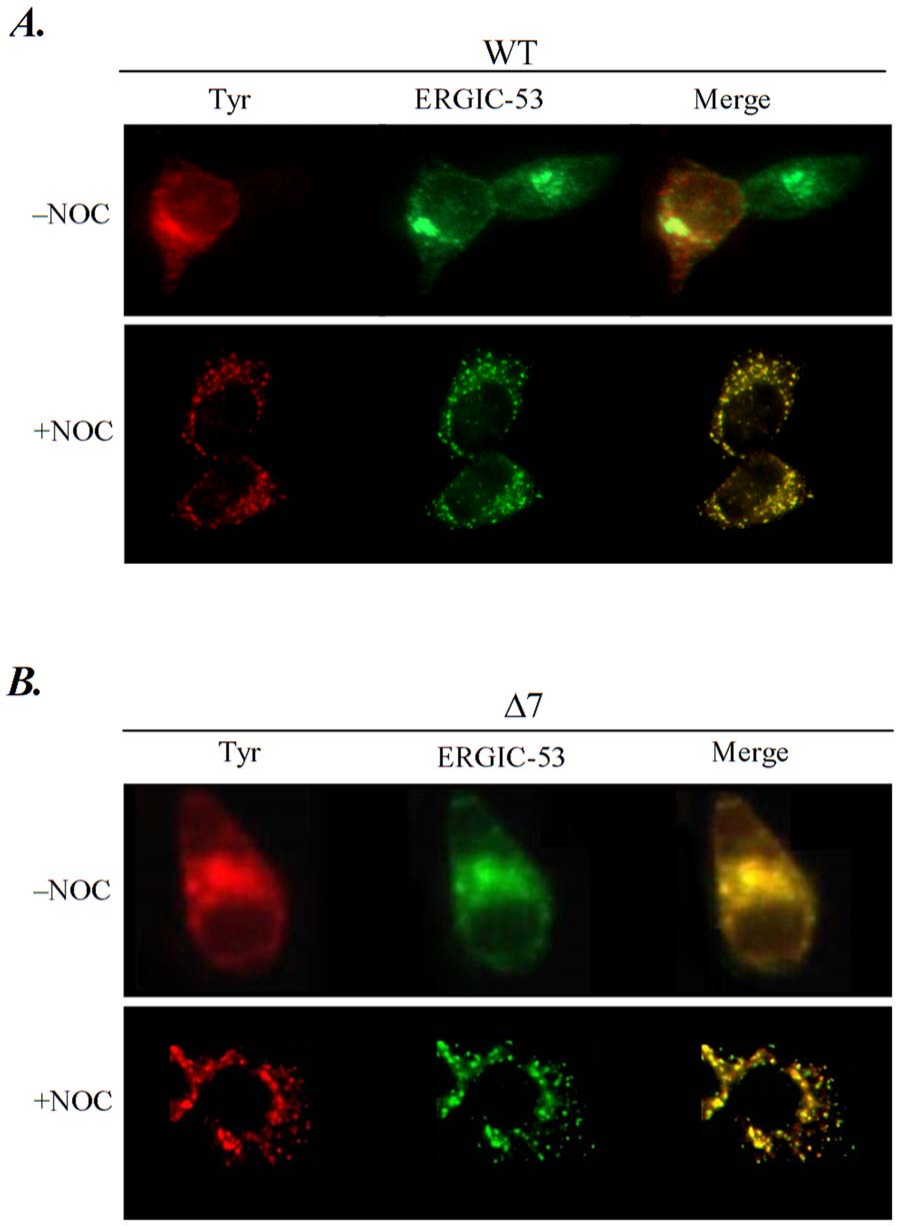
Traffic of the single N-glycosylation mutants beyond the ER in the presence of nocodazole. Cells were transiently transfected with WT and Δ7. 5 hours before immunofluorescence cells were incubated or not with nocodazole (NOC), fixed and double-labeled with anti-tyrosinase IgG2a (Tyr) and anti-ERGIC-53 IgG1 (ERGIC-53) monoclonal antibodies. Alexa Fluor 488 anti-monoclonal IgG1 and Alexa Fluor anti-monoclonal IgG2a were used as secondary antibodies. The merged images showing co-localization of tyrosinases with ERGIC-53 are also shown.

Before secretion, tyrosinase has to pass a post ER check point in the Golgi which is the decisive step towards secretion versus recycling back into the ER [50]. In contrast, some OCAI mutants are retained at the ER without further traffic to the Golgi compartment and degraded by ER associated degradation processes [23], [24]. To assess the retention process of the N-glycosylation mutants, we analyzed their co-localization with the ER-Golgi intermediate compartment (ERGIC) marker, ERGIC-53. This mannose lectin transports ER cargo glycoproteins to the ERGIC and the Golgi [51]. A typical co-localization experiment is shown in Fig. 6, for wild type tyrosinase (Fig. 6A) and the Δ7 mutant (Fig. 6B). Since ERGIC-53 cycles between the ER, the ER intermediate compartment and Golgi inhibition of the Golgi to ER pathway in the presence of nocodazole was shown to arrest the ERGIC-53 within the Golgi, with a visible change in its subcellular distribution [24], [51]. In the absence of the microtubules-based traffic the population of ERGIC-53 molecules that reached the Golgi displayed a punctuate distribution outside the ER area. Wild type tyrosinase partially co-localized with ERGIC-53 within the ER and following the nocodazole treatment could be seen co-localizing with the ERGIC-53 even outside the ER. While this confirmed previously published data, the similar Golgi pattern displayed by the Δ7 mutant in the presence of disrupted retrograde pathway indicates a transport of the misfolded mutant to the Golgi before its final re-location to the ER and degradation. All N-glycosylation tyrosinase mutants co-localized with ERGIC-53 in nocodazole treated cells in punctuate structures outside the ER, (supplemental Figure S3) indicating that the recycling pathway is common to wild type and all its N-glycosylation mutants, possibly as part of the ERAD system.

Taken together, the data show that neither the ERAD pathway, nor the interactions with EDEM1 were dependent on specific N-glycans. Incompletely folded or misfolded glycosylation mutants and wild type tyrosinase were recycled between the ER and the Golgi. However, the C-terminus glycosylation sites were more exposed to the mannosidase cleavage in the ER than the N-terminus sites. This indicates a role for the C-terminus glycans in the disposal of the misfolded tyrosinases through the mannose timer mechanism.

## Discussion

Here we show that tyrosinase individual N-glycans have distinct roles in folding and maturation depending on their spatial position relative to the active site region. The pair of N-glycans located in the proximity of the active site of human tyrosinase, s6:N337 and s7:N371, irreversibly influence its folding and degradation processes within the ER. In contrast, the other pair, s3:N161 and s4:N230, located opposite the active site promotes the association with CRT but not CNX, and their ablation results only in a reversible inhibition of enzyme activity. The spatial arrangement of these N-glycans was inferred from a 3D model of the central, active site region of human tyrosinase (aa: 142 to 450) derived from *Streptomyces castaneoglobisporus* tyrosinase with an accuracy within less than 2.5 Å rmsd. Structural analysis indicates that rather than consisting in three well separated modules: Cu A, Cys2 and Cu B the modeled region is actually formed from two globular domains, ‘extended’ Cu A and Cu B, connected by a short linker lying within aa 272 to 308. This is predicted to be flexible by all major intrinsic disorder predictors and this property may allow the two ‘extended’ copper domains to explore multiple configurations in order to generate the enzyme active site.

While the modeled region did not include the Cys1 domain with the first two N-terminal glycans s1:N86 and s2:N111, it contained the tyrosinase functional unit Cu A-linker-Cu B and the rest of 5 N-glycosylation sites. The model was probed by single and multiple N-glycosylation mutants and the assessment of their role in folding, CNX/CRT interactions, ERAD, intracellular traffic and enzyme activity.

We found that only four of the five modeled sites were actually occupied, while Asn at site s5 was not glycosylated. This is consistent with previously reported data [27] and also with the fact that s5 is not conserved in all tyrosinases. It is absent in dogs, cats, rodents and fishes, while primates and pigs have proline in position N+3. A search through the Structural Assessement of Glycosylation Sites - SAGS database [42] shows that less than 15% of structurally documented N-glycosylation sites with proline in position N+3 are occupied. This shows that site s5 is not critical and the probability to be occupied in human tyrosinase is low. Interestingly, s5 is highly conserved and it is never followed by a proline in positon (N+3) in TRP1 and TRP2, confirming the importance of this site for the other two members of the family rather than for tyrosinase itself [8].

The four occupied sites are clustered in two groups, the C-terminal N-sites s6 and s7, located close to the active site, and s3 and s4 on the opposite side of the protein. In sequence these belong to the extended Cu B and extended Cu A, respectively and have in common the fact that they are located on bends which follow or precede regular helices. This is consistent with the very frequent location of N-glycosylation sites at points where any change of the local structure of the polypeptide chain is needed. Such N-sites act as landmarks for ending or starting stretches of regular secondary structure and increase the folding efficiency [42].

On the other hand the main structural difference between the two groups is the proximity to the active site and this remarkably correlates with the ability of mutants to recover at 31°C. Both distal N-glycosylation Δ3 and Δ4 mutants are not terminally misfolded as they retain reduced enzymatic activity and the properties of temperature sensitive mutants. In contrast the absence of proximal sites s6 and s7 has far more severe effects on tyrosinase function. Not only that the activity of Δ6 and Δ7 mutants is completely lost, but very low or no recovery is found at 31°C. In addition these C-terminal sites, s6 and s7, are compulsory for tyrosinase ER maturation since in their absence tyrosinase is recycled between the ER and Golgi and eventually retained within the ER and targeted for degradation by EDEM1. The lower stringency for the presence of distal N-glycans at s3 and s4 is consistent with the conservation of these sites during evolution. For example s3 is occasionally missing in tyrosinases or TRP2s of some Salmonidae and Ictaluridae fishes, and it is always missing from TRP1s while s4 is absent in some Salmonidae fishes or even some Bovidae mammals. By contrast s6 and s7 are strictly conserved in all vertebrate tyrosinases, even though s6 is missing in TRP1 and TRP2 of some lower species. The fundamental role of s7 is also indicated by that it is located on a complex loop connecting the two helices hosting H363, H367, H390 involved copper binding. Furthermore, as seen from Fig. 5, N-site s7 is in less than 10 Å proximity to the most disordered region of the linker suggesting that this site might be critical for the quality control system in monitoring the conformational transitions in the linker region during the folding process. Naturally occurring mutations at no less than six locations on this loop, of which two ablate s7, lead to albino phenotypes, and models indicate that M374, S375 are involved in catalysis [52]. In addition the Δ7 mutant adopts a DTT sensitive conformation unlike the wild type tyrosinase, suggesting that at least some disulfide bonds remain exposed or incorrectly formed.

Tyrosinase folding starts co-translationally during the polypeptide translocation into the ER lumen. However the completion of a productive folding requires post-translational steps as previously reported [23], [25]. These consist in the tight regulation of the polypeptide chain maturation by the quality control system in which N-glycans play a central role during the entire process. Previous work focusing on the folding pathways in mouse tyrosinase N-glycosylation mutants has led to the notion that the quality control system in the ER operates locally with individual N-glycans having distinctive roles in glycoprotein folding and that chaperones associate with and dissociate from the nascent polypeptide at specific locations and for specific durations in order to ensure the correct folding of the glycoprotein [20], [21]. While CRT role in tyrosinase folding is not completely elucidated, only minutes amount of the Δ4 and Δ3 mutants associated with CRT, indicating that these two N-glycans are more accessible to this chaperone. CRT might have a different role then CNX in tyrosinase maturation, since soluble tyrosinase lacking the TM domain is unable to undertake a productive folding pathway despite its CRT association [24]. Our data are in agreement with the previously published results for another membrane glycoprotein, influenza hemmaglutinin whose N-glycans associate with CRT or CNX depending on their spatial location [4].

The first two N-terminal glycans s1:N81 and s2:N111 that remained structurally unaccounted in our model are located within the Cys1 domain, aa20–140, containing an EGF signature but also a region with high disorder propensity located in-between aa 38–53. They were shown to mediate tyrosinase co-translational folding through their interaction with CNX immediately after the removal of the signal sequence [25]. In contrast the post-translational folding requires different molecular determinants. We showed previously the importance of the TM domain in this process, related to firmly anchoring the polypeptide in the vicinity of CNX [23]. The data presented here show that in addition to TM, the C-terminal N-glycans s6:N337 and s7:N371 close in space to the active site are also strictly required to complete the post-translational folding of tyrosinase. The prolonged association with CNX/CRT of the Δ7 and Δ(5,6,7) mutants suggests a different mechanism for preventing tyrosinase exit from the ER. These oligosaccharides may not be critical CNX targets, but their deletion could impair the folding of the active site with dramatic repercussions upon the exposure of hydrophobic patches. The vital role of s6 and s7 is supported by tyrosinase pathology, since a number of mutations resulting in their ablation - S339G, N371Y, N317T, T373K – have been identified in albino phenotypes [16], [17].

All glycosylation mutants are subjected to degradation through the ERAD pathway with Δ6 and Δ7 sharing some peculiarities. The first step of the intricate timing mechanism for ERAD is the mannosidase I catalyzed cleavage of a mannose residue of proteins retained for longer periods within the ER [53], [54], [ and 55]. This is the signal for the disposal of misfolded proteins in proteasomes. Our data show that all glycosylation single mutants are targeted for degradation in proteasomes. All mutants associate with EDEM1 and accumulate in the presence of the ER mannosidase inhibitor kifunensine, indicating that the mannoses cleavage could be a key degradation signal for tyrosinase similar to other proteins [52], [56]. Importantly, the analysis of the cumulative effect of kifunensine in the triple mutants indicates that the C-terminal glycans s6 and s7 are more susceptible to mannosidase trimming than the N-terminal s1, s2, and s3 glycans. Together with the fatal misfolding caused by the knock down of s6 and s7, these results suggest that the C-terminus N-glycans drive the ERAD pathway of tyrosinase and are more readily subjected to ER mannosidase trimming.

That N-glycans do not play equivalent roles in protein processing has been shown previously for the cystic fibrosis membrane conductance regulator, a channel protein involved in the pathology of the cystic fibrosis [57]. In this case, glycans are required for calnexin and EDEM binding but not for the channel function, with the N900 glycan promoting folding and the N894 glycan supporting degradation. Another example is the yeast carboxipeptidase Y, a soluble glycoprotein with four N-linked glycans targeted through the Golgi to the yeast vacuole. Only the C-terminal N-glycan drives the polypeptide into the degradation pathway, with the N-terminal glycans involved in efficient intracellular transport [58], [59]. Finally, it has been reported that the apical sorting of the transmembrane cell adhesion molecule CD 164 was dependent of two of its eight glycans [60].

Taken together the herein data indicate that in contrast to the glycosylation of distal N-sites, the presence of N-glycans in precise conserved C-terminal location in close vicinity to the active site is critical for the correct folding of the enzyme. The absence of post-translational modification at these locations alters irreversibly the maturation pathway in a manner recognized by the quality control and degradation ER systems.

## Supporting Information

Figure S1 **The potential glycosylation site s5 is not occupied in melanoma A 375 cell line.** Transfected A375 cells were lysed and postnuclear lysates were subjected to EndoH digestion and blotted with tyrosinase antibody (T311). The Δ5 mutant acquires complex glycans resistant to EndoH and migrates at the same molecular mass as WT tyrosinase.
(TIF)

Figure S2 **Association of the Δ1, Δ2, Δ3 and Δ6 mutants with calnexin and calreticulin.** Cells were transiently transfected with Δ1, Δ2, Δ3 and Δ6 mutants. 24 h post-transfection cells were pulsed for 20 minutes and chased for 0, 30, 60, 120 min. Cell lysates were sequentially precipitated with anti-calnexin or anti-calreticulin and T311 antibodies (Ip CNX, Ip CRT). To determine the total amount of tyrosinase an aliquot of the lysate was precipitated with T311 antibodies (Ip TYR). Samples were subjected to 10% SDS PAGE and autoradiographed. One of at least two representative experiments is shown. The ratio CNX (***▴***) and CRT (***•***) bound tyrosinase/total tyrosinase over time has been plotted.
(TIF)

Figure S3 **Traffic of the single N-glycosylation mutants beyond the ER in the presence of nocodazole.** Cells were transiently transfected with Δ1–Δ6 mutants. 5 hours before immunofluorescence cells were incubated or not with nocodazole (NOC), fixed and double-labeled with anti-tyrosinase IgG2a (Tyr) and anti-ERGIC-53 IgG1 (ERGIC-53) monoclonal antibodies. Alexa Fluor 488 anti-monoclonal IgG1 and Alexa Fluor anti-monoclonal IgG2a were used as secondary antibodies. The merged images showing co-localization of tyrosinases with ERGIC-53 are also shown.
(TIF)
