# Supplementary figures and images for: C-Terminus Glycans with Critical Functional Role in the Maturation of Secretory Glycoproteins
Source: PLoS One. 2011 May 18;6(5):e19979. doi: 10.1371/journal.pone.0019979 (PMC3097235; doi:10.1371/journal.pone.0019979)

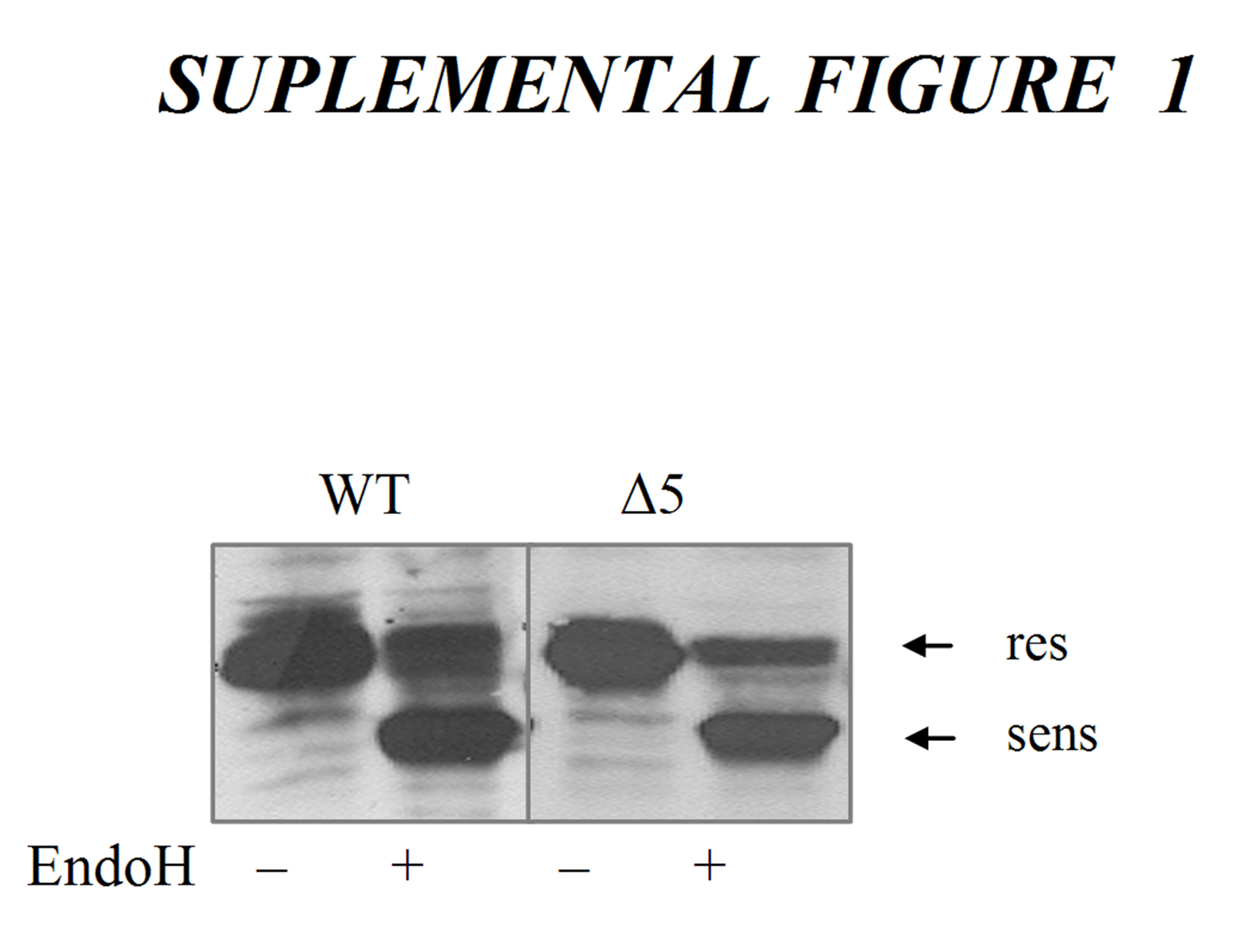

Supplement: Figure S1 — The potential glycosylation site s5 is not occupied in melanoma A 375 cell line. Transfected A375 cells were lysed and postnuclear lysates were subjected to EndoH digestion and blotted with tyrosinase antibody (T311). The Δ5 mutant acquires complex glycans resistant to EndoH and migrates at the same molecular mass as WT tyrosinase. (TIF) [file pone.0019979.s001.tif]

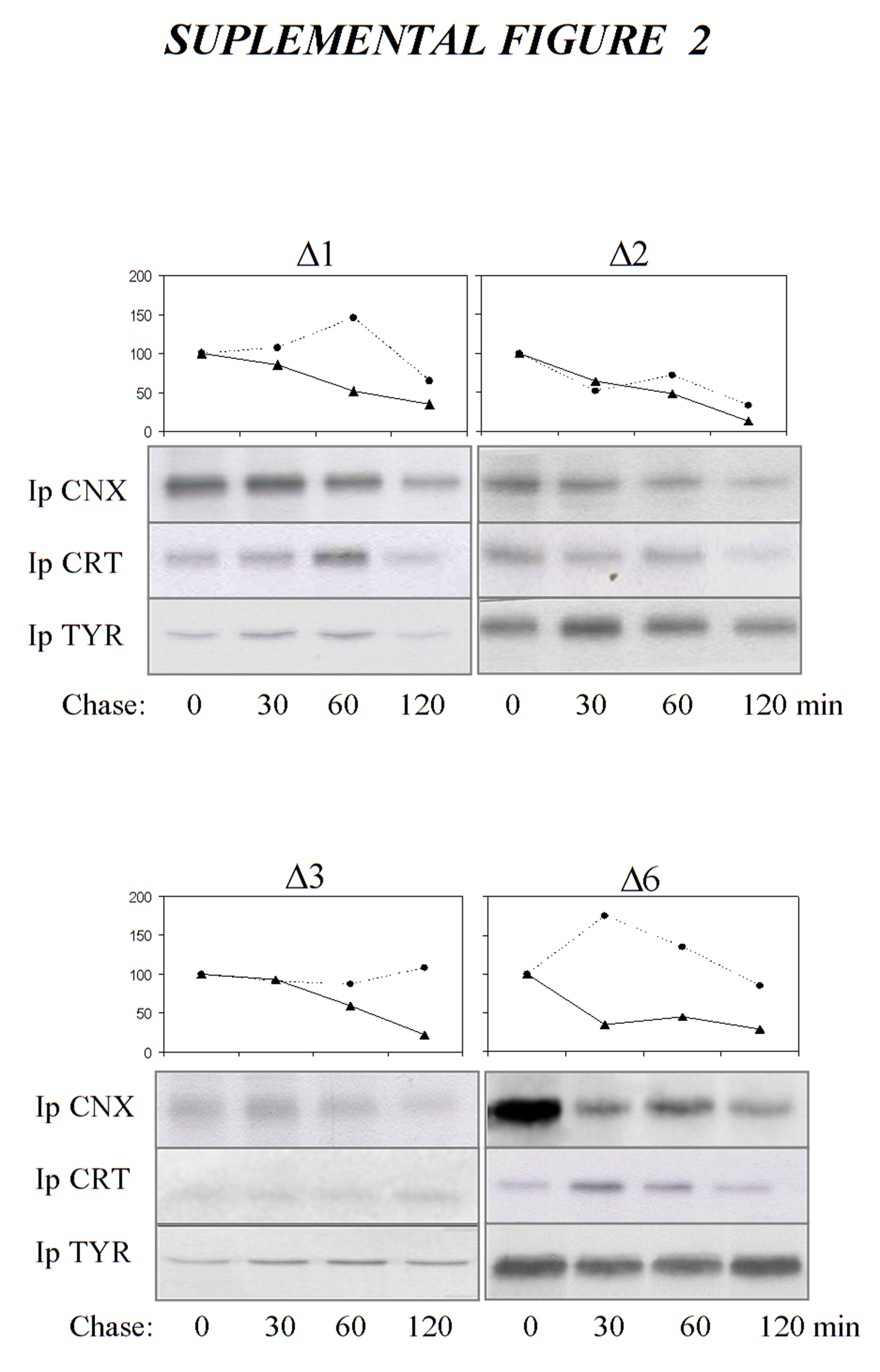

Supplement: Figure S2 — Association of the Δ1, Δ2, Δ3 and Δ6 mutants with calnexin and calreticulin. Cells were transiently transfected with Δ1, Δ2, Δ3 and Δ6 mutants. 24 h post-transfection cells were pulsed for 20 minutes and chased for 0, 30, 60, 120 min. Cell lysates were sequentially precipitated with anti-calnexin or anti-calreticulin and T311 antibodies (Ip CNX, Ip CRT). To determine the total amount of tyrosinase an aliquot of the lysate was precipitated with T311 antibodies (Ip TYR). Samples were subjected to 10% SDS PAGE and autoradiographed. One of at least two representative experiments is shown. The ratio CNX (▴) and CRT (•) bound tyrosinase/total tyrosinase over time has been plotted. (TIF) [file pone.0019979.s002.tif]

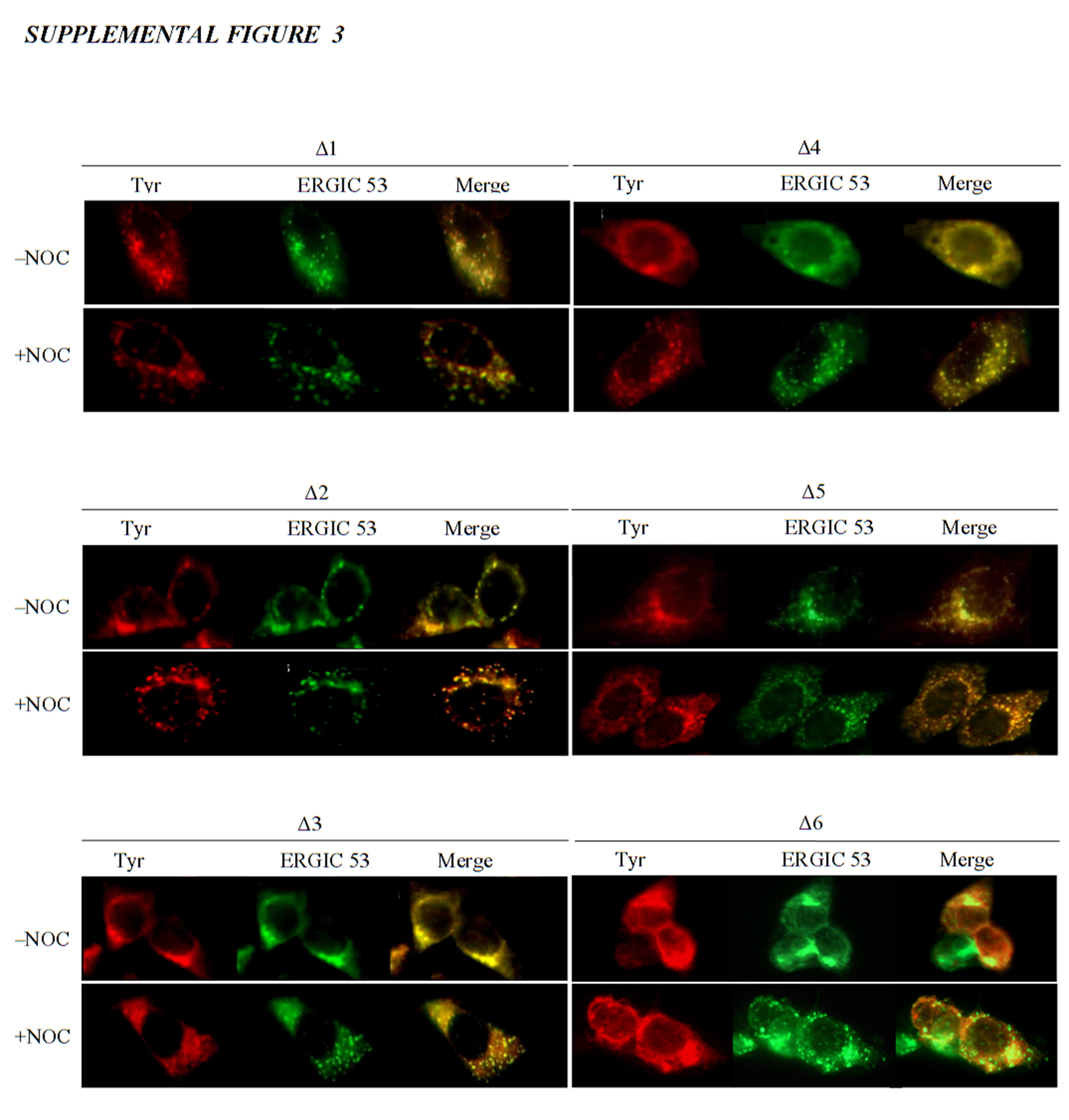

Supplement: Figure S3 — Traffic of the single N-glycosylation mutants beyond the ER in the presence of nocodazole. Cells were transiently transfected with Δ1–Δ6 mutants. 5 hours before immunofluorescence cells were incubated or not with nocodazole (NOC), fixed and double-labeled with anti-tyrosinase IgG2a (Tyr) and anti-ERGIC-53 IgG1 (ERGIC-53) monoclonal antibodies. Alexa Fluor 488 anti-monoclonal IgG1 and Alexa Fluor anti-monoclonal IgG2a were used as secondary antibodies. The merged images showing co-localization of tyrosinases with ERGIC-53 are also shown. (TIF) [file pone.0019979.s003.tif]
